# Crosstalk Between Tumor-Associated Microglia/Macrophages and CD8-Positive T Cells Plays a Key Role in Glioblastoma

**DOI:** 10.3389/fimmu.2021.650105

**Published:** 2021-07-29

**Authors:** Sheng Tu, Xu Lin, Jili Qiu, Jiaqi Zhou, Hui Wang, Shiyao Hu, Yihan Yao, Yali Wang, Yongchuan Deng, Yunxiang Zhou, Anwen Shao

**Affiliations:** ^1^State Key Laboratory for Diagnosis and Treatment of Infectious Diseases, National Clinical Research Center for Infectious Diseases, Collaborative Innovation Center for Diagnosis and Treatment of Infectious Diseases, The First Affiliated Hospital, College of Medicine, Zhejiang University, Hangzhou, China; ^2^Department of Thoracic Surgery, The First Affiliated Hospital, School of Medicine, Zhejiang University, Hangzhou, China; ^3^Department of Surgical Oncology, The Second Affiliated Hospital, School of Medicine, Zhejiang University, Hangzhou, China; ^4^Department of Medical Oncology, The Second Affiliated Hospital, School of Medicine, Zhejiang University, Hangzhou, China; ^5^School of Medicine, Zhejiang University, Hangzhou, China; ^6^Department of Neurosurgery, The Second Affiliated Hospital, School of Medicine, Zhejiang University, Hangzhou, China

**Keywords:** tumor, microglia, macrophages, CD8-positive T cells, glioblastoma

## Abstract

Glioblastoma is considered to be the most malignant disease of the central nervous system, and it is often associated with poor survival. The immune microenvironment plays a key role in the development and treatment of glioblastoma. Among the different types of immune cells, tumor-associated microglia/macrophages (TAM/Ms) and CD8-positive (CD8+) T cells are the predominant immune cells, as well as the most active ones. Current studies have suggested that interaction between TAM/Ms and CD8+ T cells have numerous potential targets that will allow them to overcome malignancy in glioblastoma. In this review, we summarize the mechanism and function of TAM/Ms and CD8+ T cells involved in glioblastoma, as well as update on the relationship and crosstalk between these two cell types, to determine whether this association alters the immune status during glioblastoma development and affects optimal treatment. We focus on the molecular factors that are crucial to this interaction, and the role that this crosstalk plays in the biological processes underlying glioblastoma treatment, particularly with regard to immune therapy. We also discuss novel therapeutic targets that can aid in resolving reticular connections between TAM/Ms and CD8+ T cells, including depletion and reprogramming TAM/Ms and novel TAM/Ms-CD8+ T cell cofactors with potential translational usage. In addition, we highlight the challenges and discuss future perspectives of this crosstalk between TAM/Ms and CD8+ T cells.

## Introduction

Glioblastoma, also known as glioblastoma multiforme (GBM), is one of the most malignant diseases that is thought to originate within the central nervous system (CNS) (1). Although significant progress has been made in various treatments, such as chemotherapy, radiotherapy and surgery, the survival rate among glioblastoma patients has not shown progressive improvement using these conventional treatments (2–4). In the past few decades, there have been significant new therapeutic advances in oncology, wherein small molecules have shown promising curative effects among many types of malignant tumors. However, given the special anatomic structure of the CNS, many therapeutic molecules are not able to reach the target zone because of the blood–brain barrier (BBB), which is a highly selective barrier in the CNS (5), leading to poor survival and prognosis of patients with malignant CNS tumors.

Nonetheless, recently, immune therapy has shown promising outcomes (6–9) by overcoming the selectivity of BBB as immune cells are highly infiltrative in the tumor microenvironment of glioblastomas (10). The immune system in the CNS plays a significant role in GBM development (11). The tumor microenvironment of GBM is infiltrated with various types of immune cells and cytokines. CD8-positive (CD8+) T cells are the most important type of immune cells in immune therapy as they function as tumor cell killers. Furthermore, macrophages are the most infiltrated immune cells (12, 13) in GBM. Hence, tumor-associated macrophages (TAMs) account for the majority of macrophages within GBM, and are recruited from circulation. Most TAMs are considered to be immunosuppressive agents that are associated with tumor immune escape, as well as angiogenesis and invasion. Microglia are resident immune cells of the CNS that interact as housekeepers of the CNS and participate in the innate immune responses and antigen presentation. However, microglia are silenced in the GBM microenvironment and support immunosuppression. Given their common biological functions, microglia and macrophages in GBM tumors (as in other tumors) are known as tumor-associated microglia/macrophages (TAM/Ms) (14, 15). The crosslink between GBM-associated immune cells and TAM/Ms and CD8+ T cells, which show great potential given their direct and indirect contact with GBM cells in the immune response, can serve as a potential therapeutic target for GBM.

Herein, we outline the physiological, pathological, and micro-environment features of TAM/Ms and CD8+ T cells with regard to GBM development, as well as the impact of their crosstalk on tumor malignancy, and a potential translational scheme for glioblastoma treatment.

## Role of TAM/Ms in Glioblastoma

Macrophages and microglia have a significant function in the innate immune response of a healthy brain tissue, such as in anti-inflammatory and scavenger processes (14). Microglia and macrophages are classified as neuronal support cells that are located between nerve fibers, and function to carry out the primary immune responses of the nervous system as killer cells and phagocytes. Microglia originate from the embryonic mesoderm and are located in the nerve tissue; likewise, some macrophages develop in a similar manner. However, macrophages can also be derived from monocytes that migrate from circulation to become adult macrophages (14).

In the physiological innate response of the nervous system, macrophages and microglia recognize pathogens or eliminate cells through the pathogen-associated molecular patterns (PAMPs) and damage-associated molecular patterns (DAMPs). These activate a cascade of immune response reactions that function against infection, ischemia, trauma, or other threats. As oligomeric protein complexes are activated in response to PAMPs and DAMPs, inflammasomes activate and release inflammatory cytokines, including IL-1β and IL-18. Various inflammasomes, inflammatory cytokines, and chemokines play contrasting roles with regard to cancer development and progression. Macrophages and microglia are classified into M1 and M2 clusters that represent pro-inflammatory and anti-inflammatory components, respectively. Pro-inflammatory cytokines are commonly categorized in relation to the M1 phenotype, and include interferon-gamma (IFN-γ), tumor necrosis factor-α (TNF-α), IL-2, and IL-12. In contrast, immunosuppressive molecules, such as IL-4, IL-10, IL-13, and tumor growth factor-β (TGF-β), are classified in relation to the M2 phenotype (16–19). Notably, within the tumor microenvironment, TGF-β plays crucial roles with regard to tumor immunity, and targeted TGF-β signaling blockade in helper T cells elicits an effective tissue-level cancer defense response that can provide a basis for therapies that are directed toward the cancer microenvironment (20); TGF-β is also the most potent immunosuppressor against cancer cells, and this significant effect is mediated in multiple ways, including polarization of macrophages to M2 cells. Additionally, several previous studies have indicated that anti-TGF-β exerts a potential and promising effect on tumor immunity (21–24). The relative literature on TGF-β indicates that TGF-β likely also plays an important role in the regulation of pathological processes in GBM patients. Moreover, a therapy targeting TGF-β can serve as a potential strategy in GBM treatment in the future. Recently, additional biomarkers have been described to play a role in macrophage polarization. TNF-α, IL-12, and IL-23 have all been confirmed to be related to the M1 phenotype (14, 25). Inducible NO synthase (iNOs) has been shown to be associated with M2 clusters in GBM cells (26). Together with a certain polarization proportion, M1 and M2 clusters of macrophages and microglia help maintain CNS homeostasis. Although M1 and M2 phenotypes are different owing to levels of differentiation, they have plasticity to a certain extent, and TAM/Ms show overlapped proportion between M1 and M2 clusters (27, 28).

With regard to GBM, TAM/Ms have common biomarkers, e.g., cluster of differentiation 68 (CD68), which is also the most commonly expressed protein in various malignant diseases (29). Nevertheless, CD163, which is highly specific for M2 macrophages, is also highly expressed in GBM cells (29–31), which suggests a dominance of the M2 cluster of TAM/Ms in the GBM tissue. In an early study on GBM-related macrophages, M1 and M2 clusters were classified together as being histologically similar to each other. Studies have indicated that the inclusion of TAM/Ms into the M2 family is responsible for their non-phagocytic function and anti-immune responses in GBM; TAM/Ms are further classified into M2a, M2b, and M2c subtypes based on their different roles in GBM response. However, specific exclusive characteristics of the M2 family between categories remains to be further elucidated (15). The M1 and M2 polarity classification is defined in the inflammatory response. However, TAM/Ms in GBM play a role in the carcinogenesis process. Differences in the origin of macrophages and activation of the innate immune response induces different responses, dependent on the microenvironment, helping the formation of heterogeneous communities and subgroups with appropriate functions. In the GBM microenvironment, the predominant activity of TAM/Ms takes place through the M2 phenotype, whereas the immune status of TAM/Ms favors immune suppression. Moreover, the enrichment of the M2 cluster TAM/Ms is associated with the prognosis and survival of GBM patients (32–34). From a biochemistry perspective, GBM cells produce immunosuppressive cytokines, including IL-10 (35), macrophage-inducing chemokine monocyte chemotactic protein-1 (CCL2) (16), and cell-surface protein colony-stimulating factor 1 receptor (CSF1R) (36). Transcriptomic analysis of GBM cells has demonstrated a positive relationship between invasion-related genes and immunosuppressive genes (37, 38). Hence, polarized TAM/Ms have been reported to stimulate carcinogenesis *via* their immune-escape effect and immunosuppressive secretions. Further, cancer growth factors have also been identified in GBM cells co-cultured with TAM/Ms (25). With the help of these growth factors, GBM cells are able to suppress TAM/M polarization into the M1 phenotype and induce polarization into the M2 phenotype by recruiting and re-differentiating macrophages into M2-dominant clusters, thus creating a pro-carcinogenesis immunosuppressive environment.

Previous studies have validated that TAM/Ms are related to the prognosis/survival outcomes of GBM patients. One previous study (39) used automated quantitative immunofluorescence to identify the prognostic impact of TAMs in GBM. The results of their study suggested that M2-like TAMs hold an unfavorable prognostic value in high-grade gliomas. Similarly, Pimenta et al. (40) reported that untreated GBM patients with TAM infiltration demonstrated a shorter overall survival compared with the patients without TAM infiltration. Additionally, the number of microglia/macrophages with positive staining for CD163 and CD204, which are thought to be markers for M2 macrophages, was found to be correlated to the histological grade of gliomas, whereas the ratio of M2 macrophages in the TAM/Ms was related to the histological grade (41). This suggests that evaluation of the proportion of M2 microglia/macrophages in GBM is useful for the assessment of the prognosis of patients with gliomas, including GBM. Kaffes et al. (42) determined that high expression of the TAM-related gene *AIF1*, which encodes the TAM-specific protein IBA1, is correlated with a worse prognosis in pro-neural GBM, but confers a survival benefit in mesenchymal tumors. Recently, numerous studies have reported that TAM/Ms regulation may be a potential therapy strategy in cases with GBM. A new report demonstrated that PI3Kγ inhibition could suppress TAM/M accumulation in glioblastoma microenvironment, and enhance the anti-neoplastic effects of temozolomide in glioblastoma cells (43). In addition, the deletion of HuR (an RNA regulator) in TAM/Ms could attenuate glioma growth (44). Further, Martins et al. provide a comprehensive overview of microglia-centered combinatorial strategies against glioblastoma, and conclude that MG modulation, as a central paradigm in GBM immune tumor microenvironment, may lead to additional, long-lasting, and effective tumoricidal responses (45).

Altogether, TAM/Ms appear to be involved in the development of GBM *via* atypical polarization and by creating an immunosuppressive microenvironment, and are tightly associated with the outcome of GBM patients. Thus, we can infer that TAM/M polarization, recruitment, and function of immunosuppressive cytokines may be potential targets in GBM treatment.

## Role of CD8-Positive T Cells in Glioblastoma

CD8+ T cells are a subtype of T cells that develop within the thymus and have a cytotoxic effect on cancer cells through antigen recognition using major histocompatibility complex class I (MHC-1) (46). In cases of carcinoma, CD8+ T cells are characterized to be able to kill cancer cells directly at the end of their cancer immune response at any phase of malignancy.

There are three steps involved in the differentiation of naïve T cells into cytotoxic CD8+ T cells, which leads to a cytotoxic effect on GBM and other cancers. First, the immunogenic antigens that are aberrantly expressed are processed through antigen-presenting cells, such as dendritic cells (DC), and presented to naïve T cells at peripheral lymphatic sites, such as lymph nodes. Activated CD8+ T cells then go on to proliferate clonally and migrate to the GBM site, where CD8+ T cells recognize tumor cells through MHC-1 and release cytotoxic signals that initiate the anti-tumor effect. Traditionally, the central nervous system is considered to be an immune-exempted organ. Recently, many studies have identified that under pathological conditions, the integrity of the BBB and the blood–cerebrospinal fluid barrier are compromised, causing inflammatory cells to enter the brain and activate the immune system (47, 48). However, CD8+ T cell infiltration varies widely between different types of GBMs. An *in vivo* study demonstrated a substantially increased infiltration of CD8+ T cells in mesenchymal GBM, whereas immune infiltrates were rarely found in pro-neural GBM. Furthermore, the molecular subtype of cancer stem cells (CSCs) TGF-β-dependently contributed to the degree of immune infiltration in patients with GBM (49). Cancer vaccination has been developed to amplify this anti-cancer cytotoxic effect by CD8+ cells (9). However, glioblastoma can escape from natural immune response by immune inhibition. To overcome this challenge, immune checkpoint therapy has been developed that can help boost anti-cancer immune response (6). However, in some cases, GBM may undergo immune escape by not presenting tumor antigens or MHC-1, thus preventing recognition by CD8+ T cells. Genetic modification is another promising method that involves adaptively transferring target antigens to CD8+ T cells and cultivating them clonally. These CD8+ T cells, known as chimeric antigen receptor T cells (CAR-T), have the ability to recognize immune-escaped tumor cells (50, 51).

Various studies have assessed the importance of CD8+ T cells with regard to outcomes of GBM patients. Audencel is a dendritic cell (DC)-based cellular cancer immunotherapy against GBM. Although the recent phase II “GBM-Vax” trial shows no clinical efficacy of Audencel, as assessed through progression-free and overall survival of GBM patients, post-vaccination levels of CD8+ T cells in the blood were indicative of a significantly better survival among GBM patients (52). A report by Yang et al. (53), which included 519 GBM patients, suggested that tumors from long-term survivors, more likely than those form short-term survivors, have either intermediate or extensive CD8+ T cells infiltrates compared with focal or rare infiltrates. This indicates that prolific CD8+ T cell infiltrates appear to correlate with partitioned long-term survival among newly diagnosed GBM patients. Also, increased CD8+ to FoxP3+ regulatory T cell ratios showed a positive correlation with survival outcomes in primary GBM, indicating that absolute numbers of CD8+ T-cells and effective balance of CD8+ T cells to FoxP3+ regulatory cells are both informative for predicting clinical outcomes in patients with GBM (54).

## Crosstalk Between Tumor-Associated Microglia/Macrophages and CD8+ T Cells

During cancer development, tumor tissue is infiltrated by various immune response cells that can be classified into either immune-effector cells or immune-supporting cells. Among tumor-associated immune cells in GBM, CD8+ T cells are known to be major cytotoxic lymphocytes (CTLs) and TAM/Ms are the most infiltrated immune-supporting cells (55).

The dominant effect of TAM/Ms in GBM is immunosuppression, which is manifested through the expression of PD-L1 and PD-L2, as well as the CTL-associated protein 4 (CTLA-4) ligands CD80 and CD86. The main target of these immune checkpoints is CD8+ T cells, which leads to the inhibition of its cytotoxic effects. To expand into the therapeutic scenario, as in the case of immune checkpoint therapy, there are still several patients who do not respond to immune checkpoint inhibitors (8, 56). An ideal theoretical explanation for this may be that CD8+ T cells need to overcome an intrinsic negative regulation of the immune system. In cases of GBM, TAM/M-induced immunosuppression is one of the major reasons for passive response toward immune checkpoint therapy. *In vivo* studies have determined that TGF-β secreted by TAM/Ms inhibits T cell-mediated tumor clearance (57). Next, TGF-β targets proteins that are responsible for CTL-mediated tumor cytotoxicity, including perforin, granzyme A, granzyme B, first apoptosis signal ligand (FASL), and IFN-γ. While IFN-γ is the main trigger of cytotoxic activity, FASL is the key element that activates apoptosis of target cells. Neutralization of TGF-β through an antagonist in a mouse model has been shown to upregulate cytotoxic gene expression among CD8+ T cells (57). In contrast, TAM/M-originated TGF-β also causes differentiation of naïve T cells to regulatory T (Treg) cells, which is another CD8+ suppressor in the tumor microenvironment (58). Moreover, IL-10 secretion by TAM/Ms also stimulates Treg differentiation (59). A study by Takenaka et al. (60) reported that kynurenine produced by GBM cells activates aryl hydrocarbon receptor (AHR) in TAMs, which helps modulate their function and T-cell immunity. AHR drives the expression of the ectonucleotidase CD39 in TAMs, which promotes CD8+ T cell dysfunction by producing adenosine in conjunction with CD73, AHR, and CD39 expressed in TAMs. This participates in the regulation of the immune response, including CD8+ T cell regulation in GBM, and constitutes potential targets for immunotherapy.

TAM/Ms also play a significant role with regard to infiltration of CD8+ T cells in malignant tumors. Fibrosis is a major target for TAM/Ms to regulate CD8+ T cell migration (61, 62), led by extracellular matrix stiffness and collagen deposition. CCL2, produced in the glioma microenvironment, is essential for the recruitment of regulatory T cells and myeloid-derived suppressor cells (63), as well as macrophages that are dependent on colony stimulating factor-1 (CSF-1) for both differentiation and survival (64). Therefore, TAM depletion *via* CCL2 or CSF-1 inhibitors have been reported to increase CD8+ T cell migration and infiltration (65) as it helps overcome immunosuppression.

In addition, TAM/Ms are able to regulate T cell metabolism. Arginase belongs to a family of L-arginine enzymes and regulates arginine metabolism. Arginase overexpression by TAM/Ms leads to metabolic starvation of T cells through indoleamine 2,3-dioxygenase (IDO) pathway (59, 66).

Moreover, in melanoma and pancreatic cancer, TAM/Ms are able to intervene in CD8+ T cell function by expressing V-domain Ig suppressor of T cell activation (VISTA), which causes a suppressive CD8+ T cell response (67).

**Figure 1:**
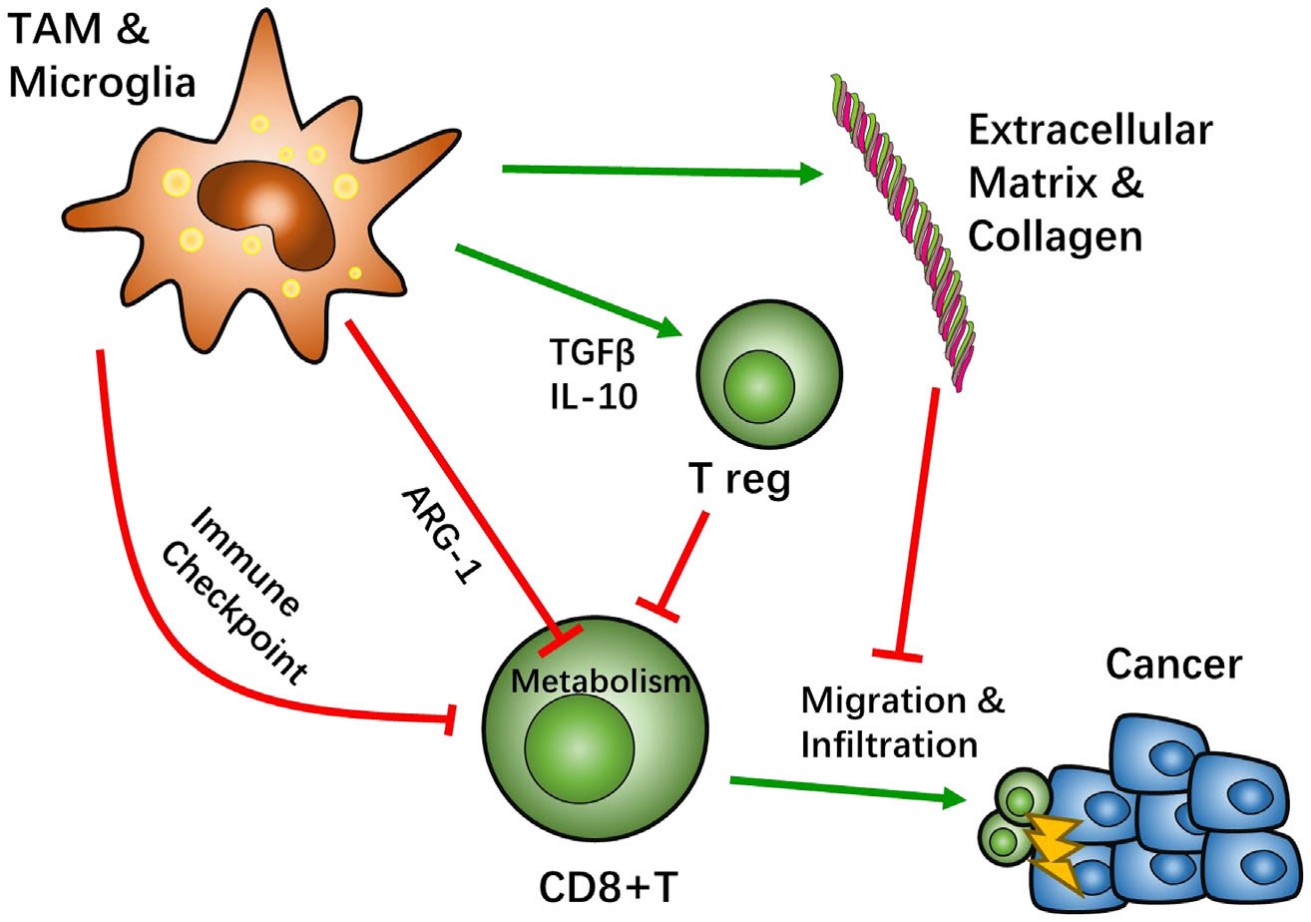
Crosstalk between tumor associated macrophages (TAMs) and CD8-positive (CD8+) T cells. Most TAMs function as tumor suppressive agents. TAMs create an immunosuppressive microenvironment through a sophisticated network. The classical pathway to inhibit CD8+ T cell function is through immune checkpoint recognition. TAMs present immune inhibition signals, such as programed cell death protein (PD-1) pathway or cytotoxic T-lymphocyte-associated protein 4 (CTLA4) pathways, which hamper CD8+ activation. TAMs also recruit other immunosuppressive cells, such as regulatory T cells (Treg), by secreting IL-10 and TGFβ to negatively regulate CD8+ T cells. Studies suggest that the metabolism of CD8+ T cells can be affected by TAMs. Specifically, TAMs can lead T cell starvation though arginine metabolism. After activation of CD8+ T cells, TAMs can help influence the migration and infiltration of CD8+ T cells through extracellular matrix stiffness and collagen deposition to have an effect on cytotoxicity.

Interactions between TAM/Ms and CD8+ T cells are known to be involved across all stages of GBM development, including infiltration, differentiation, and tumor cell interaction. This complexity of the immune microenvironment interplay causes the heterogeneity of GBM tissue across different patients. Based on this, there are many promising treatment targets for GBM that are likely helpful for patients who cannot benefit from the current standard treatment of GBM (**Figure 1**).

## Novel Therapeutics Based on TAM/M Regulation for Enhancing Effector T Cells in Glioblastoma

Given their physiology, TAM/Ms do not have the characteristic of eliminating tumor cells directly. Individually, the ideal role of CD8+ T cells in tumor tissue is to carry out cytotoxic activity against malignant cells. Likewise, there are a few novel therapies that have tried to reverse the process of innate and adaptive immune responses that are suppressed by the tumor microenvironment, either through direct or indirect schemes. This includes immune checkpoint inhibitor (ICI) or chimeric antigen receptor T cell (CAR-T) therapy. Several ongoing clinical trials have depicted great progress in GBM treatment with ICI (56, 68) and CAR-T therapy (69, 70). However, a large number of patients still do not respond to these treatments (71), largely because of the failure of CD8+ T cells to overcome the immune inhibitory microenvironment. TAM/Ms are key cells that generate and maintain this immunosuppressive environment in GBM. There are potential treatments for GBM that underlie this crosstalk between the immune effector CD8+ T cells and the immune supporter TAM/Ms. Finally, a majority of therapies that target the regulation of TAM/Ms for enhancing effector T-cell function in GBM are based on three methods, i.e., TAM/M depletion, TAM/M reprogramming, and other novel effective biomarkers in TAM functioning.

### TAM/M Depletion

To participate in the immune activity within the GBM microenvironment, TAM/Ms and other immune cells migrate and gather at the tumor site from the other tissues that are in the vicinity. As differentiated TAM/Ms do not have proliferative ability, a continuous supply of TAM/Ms is needed within the GBM microenvironment. Accordingly, activation of the CC-chemokine receptor 2 (CCR2) signal, which is located on the surface of TAM/Ms, induces the accumulation of TAM/Ms at the target site (72–75). It has been reported that astrocytes are the main cell types that express CCR2 in normal brain tissues, which helps maintain certain infiltration of macrophages and microglia to execute an innate immune response, and maintain homeostasis in the CNS environment (73, 76). GBM cells have been reported to express the CCR2 ligand to raise microglia (16), which likely enhances GBM malignancy by creating an anti-immune environment. In a mouse model of breast cancer, a CCR2 antagonist was reported to dramatically reduce macrocyte infiltration (74). The colony-stimulating factor 1 receptor (CSF1R) is also recognized to be a TAM/M recruiting signal (77). Treatment with CSF1R antagonist PLX3397 was shown to markedly reduce the number of TAM/Ms in several cancer tissues across mouse models (36, 78, 79). There are other potential targets, including the CX3CL1/CX3CR1 complex, that elicit TAM/M adhesion and migration *via* matrix degradation through its downstream enzyme matrix metalloproteinase 2 (MMP2), which, in turn, induces GBM invasion (80). MMP9 and MMP19 also participate in this process. CXCR4 antagonists also show an inhibition of TAM/Ms’ migration in GBM, though their main target is myeloid-originated macrophages (81).

With regard to immune checkpoint therapy, TAM/M-induced immunosuppressive environment causes dormancy of CD8+ T cells. TAM/M recruitment is one of the targets that helps revive the anti-tumor activity induced by immune checkpoint inhibitors. In a mouse with pancreatic cancer, using a CCR2 antagonist was associated with significant inhibition of macrophage infiltration into the tumor region (82). Furthermore, upon combination treatment with CCR2 antagonist and the immune checkpoint inhibitor PD-1 antibody, suppression of tumor growth was found to be more effective compared with the PD-1 antibody alone. This result suggests that TAM/M recruitment is a potential target for GBM treatment as GBM is a type of TAM/M-enriched malignant cancer. In contrast, in another study, overexpression of CCR2 in GBM aggravated microglia recruitment, which induced an invasive transition of GBM cells (76).

**Figure 2:**
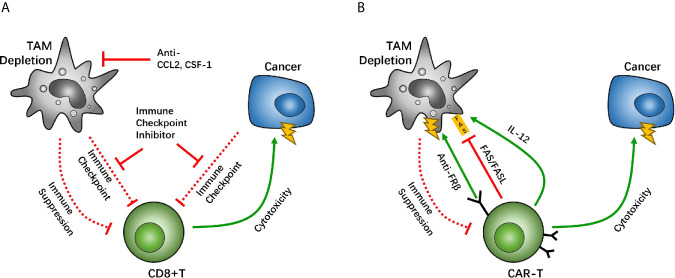
Macrophage depletion is a potential target between tumor associated macrophages (TAMs) and CD8-positive (CD8+) T cells, which holds back the immunosuppressive function of TAMs. The CC-chemokine receptor 2 (CCR2) and colony-stimulating factor 1 receptor (CSF1R) are key factors for macrophage recruitment and migration. Studies have suggested that inhibition of TAM recruitment enhances the efficacy of immune checkpoint therapy **(A).** Other studies have suggested that the chimeric antigen receptor T (CAR-T) cell overexpresses CAR-T cells with IL-12, causing TAM death through the FAS/FASL pathway, and anti-folate receptor β (FRβ) CAR-T cells have lethality toward TAMs **(B)**.

Treatment with the CSF1R antagonist PLX3397 also helps boost the efficacy of immune checkpoint inhibitors. Treatment with PLX3397, as well as the PD-1 antibody, depicted complete tumor growth blockage among mice with pancreatic cancer, whereas PD-1 antibody treatment alone demonstrated limited tumor inhibition (**Figure 2A**). Notably, an inhibitor of CSF1R that targets TAMs in a mouse pro-neural GBM model was studied, which demonstrated significantly increased survival and regression of established tumors, without TAM depletion in treated mice, thus identifying the therapeutic potential of CSF1R inhibition in GBM (64). As a negative regulator of anti-tumor immunity, TGF-β impairs the efficacy of anti-PD-1/PD-L1, and stimulates drug resistance. YM101 can help simultaneously block TGF-β and PD-L1 pathways and has a superior anti-tumor effect, compared with the monotherapies (21). In addition, the agent bintrafusp alfa is able to bring the TGF-β trap to the tumor microenvironment through its anti-PD-L1 component, which simultaneously attacks both the immunosuppressive PD-L1 and TGF-β entities, resulting in anti-tumor effect (83). A report by Zhou et al. indicated that an immunosuppressive subtype of tumor featured high immune infiltration, stromal enrichment, and TGF-β signaling pathway activation, which is likely suitable for anti-PD-L1 and anti-TGF-β combined therapy (84).

In CAR-T therapy, a decrease in the numbers of TAM/Ms also demonstrates potency to reverse anti-immune status. IL-12, a cytokine that helps regulate innate immune cells, including macrophages and NK cells, can promote anti-tumor activity effectively. When IL-12 is co-expressed in CAR-T cells with neoantigens, it allows CAR-T cells the ability to regulate TAM/Ms through its feedback mechanisms. Agliardi et al. found that local delivery of IL-12 may be an effective adjuvant for CAR-T cell therapy for GBM (85). Also, *in vivo* and *in vitro* experiments have confirmed that simultaneous use of recombinant IL-12 can increase the anti-tumor activity of CAR-T cells, especially for treatments of several types of solid tumors (86).

In an ovarian cancer study, CAR-T cells that allow the identification of tumor neoantigens were modified to secrete IL-12. The survival of patients was prolonged among those treated with the modified CAR-T cells, compared with the group without IL-12 secretion (87). In this case, IL-12 secretion is more likely to be autocrine, as demonstrated in studies with IL-12 receptor-knockout mice (87). On further evaluating the mechanisms underlying IL-12-related CAR-T-boosting effect, IL-12 overexpression in CAR-T cells demonstrated that an increase in the first apoptosis signal ligand (FASL), and thus an increase in the recognition of the first apoptosis signal (FAS) in macrophages, induces TAM/M apoptosis and thereby causes depletion of TAM/Ms (87). These results suggest that the modification of IL-12 expression can cause TAM/M depletion through the FAS/FASL pathway. Moreover, a negative feedback loop develops between CAR-T and TAM/Ms to maintain regulated immune homeostasis, which helps maximize the anti-tumor effect of CAR-T. Moreover, overexpression of IL-12 can help stimulate the cytotoxic effect of CD8+ T cells, which helps further boost the therapeutic effect of CAR-T cells (88) (**Figure 2B**). Another research team has reported that adding another TAM CAR target on folate receptor β (FRβ), which is highly expressed on TAM/Ms, can cause TAM/Ms to be eliminated in ovarian tumors and tumor growth may be delayed (**Figure 2B**). However, more studies are needed to develop CAR-T cell lines with two CAR systems on it. These studies suggest that TAM/Ms should be paid attention to during CAR-T cell treatment and co-treatment targeting TAM/Ms can help boost the effect of CAR-T therapy. Furthermore, there are other drugs that are designed for cardiovascular diseases, infections, and osteoporosis, namely telmisartan, minocycline, and zoledronic, respectively. These have been demonstrated to interfere with CCL2 synthesis, and therefore, may be associated with GBM treatment with a noncytotoxic regimen (89).

### TAM/M Reprogramming

Although activated TAM/Ms are in the end stage of differentiation, they maintain their plasticity to reprogram their characteristics, which is a hallmark of macrophages. As an example, M2 macrophages are able to shift their phenotype to M1 when they are cultured in an inflammatory signal-enriched environment, such as with lipopolysaccharides and IFN-γ (90). As the GBM microenvironment exhibits a pro-M2 phenotype, which is predominantly immunosuppressive, reprogramming TAM/M differentiation to a pro-inflammatory status may be a possible mechanism with which to re-activate suppressed CD8+ T cells.

Failure of the immune checkpoint inhibitor (ICPi) to treat GBM underscores the need for improving the therapeutic strategy, and changing TAM from the M2 type (anti-inflammatory) to M1 (pro-inflammatory) type. This helps increase the therapeutic response of ICPi, and therefore, may be a promising therapeutic strategy. Hsu et al. (91) determined that combining rapamycin (R) and hydroxychloroquine (Q) preferentially induces M2 cell death, and *in vitro* RQ treatment decreases macrophage polarization of M2, whereas a combination of RQ and anti-PD1 treatment was found to be synergistic in action, and enhanced the intra-tumoral M1/M2 ratio. This provides a rationale for manipulating the TAM phenotype, as well as increasing the therapeutic effect of ICPi in GBM.

**Figure 3:**
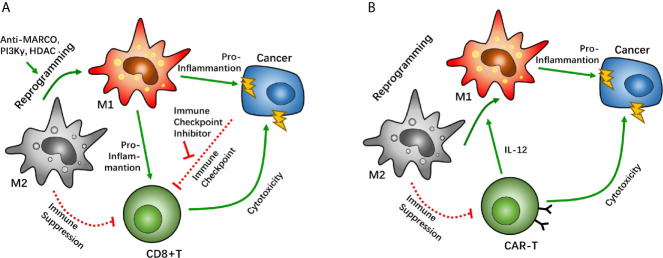
Macrophage reprogramming as a potential target between tumor associated macrophages (TAMs) and CD8-positive (CD8+) T cells. Macrophage reprogramming of TAMs can help shift the microenvironment from an immunosuppressive phenotype to a pro-inflammatory phenotype. In immune checkpoint therapy, macrophage receptors with a collagenous structure (MARCO), phosphoinositide 3-kinase γ (PI3Kγ), and histone deacetylase have been proven as targets that enhance the efficacy of immune checkpoint inhibitors through macrophage reprogramming **(A).** In chimeric antigen receptor T cell (CAR-T) therapy, CAR-T cells can reprogram TAMs by overexpressing IL-12 to boost its cytotoxicity **(B)**.

The macrophage-specialized pattern receptor macrophage receptor with collagenous structure (MARCO) is known to be specifically expressed in TAM/Ms. Recognition of the MARCO target pattern leads to reprograming of the suppressive phenotype of TAM/Ms (92). Studies have determined that MACRO-targeted antibodies can help intensify the efficacy of CTL4 antibody treatment, and thus enhance the power of immune checkpoint therapy that helps suppress tumor growth in the breast, colon, and skin. This helps reprogram macrophages against immunosuppression, which plays a role in boosting the anti-cancer ability of CD8+ T cells (92). The anti-MACRO antibody treatment reduces IL-10 expression, which helps trigger a decline in IL-1β. These cascaded cytokine fluctuations cause the TAM/M phenotype to shift from the immunosuppressive subtype to the immunostimulant subtype, thereby changing the tumor microenvironment to a pro-inflammatory phase and promoting the cytosis effect of CD8+ T cells in immune checkpoint inhibitor therapy. Phosphoinositide 3-kinase γ (PI3Kγ), a common differentiation regulator signal, also has a vital function in macrophage polarization. A previous study reported that treatment with the PD-L1 antibody significantly increased the infiltration of CD163+ macrophages in an *in vivo* GBM mouse model (31). However, the combined treatment with PD-L1 antibody and PI3Kγ inhibitor reversed the predominance of CD163, which helped boost PD-L1 antibody treatment. Immune checkpoint therapy, combined with macrophage reprograming, can help break the negative feedback induced by PD-1/PD-L1 treatment. Furthermore, treatment with PI3Kγ inhibitor reduced IL-10 and ARG1 expression in macrophages, as well as TGF-β, which enhanced the cytotoxicity of CD8+ T cells in a Lewis lung carcinoma model (93). These results suggest that the PI3Kγ inhibitor can help boost the efficacy of CD8+ T cells by reprogramming TAM/Ms and reshaping the immunosuppressive environment. Similar results have been reported in head and neck cancers as well (93). TMP195, a IIa histone deacetylase antagonist, has been proven to alter macrophage polarization from a tumor-suppressive phenotype to a classical scavenger phenotype (94). Further investigation suggested that co-treatment with the PD-1 antibody and TMP195 demonstrates improved tumor inhibition in a mouse breast cancer model (**Figure 3A**).

In CAR-T therapy, T cells are sometimes suppressed *via* a supportive anti-immune environment, in which TAM/Ms have a major role in GBM. In a mouse tumor model with CEA overexpression, CEA-CAR-T cells demonstrated anti-cancer effects after CAR-T cell injection. However, the degree of cytotoxicity was similar to when the same CAR-T cells were injected into mice with a non-CEA-related cancer. Interestingly, co-expression of IL-12 on CEA-CAR-T cells significantly enhanced the tumor-killing effect of CAR-T cells in a CEA-dependent cancer. A similar effect of IL-12 co-expression was also found in a non-CEA-related cancer (95). It has been suggested that there are underlying IL-12-related factors that help boost the cytotoxic effect of CAR-T cells. Further investigation has demonstrated that IL-12 overexpression increases macrophage infiltration. Unlike classical TAM/Ms, IL-12-dependent macrophages are identical to the pro-inflammatory phenotype, which alters the immunosuppressive microenvironment induced by the predominance of the M2 phenotype macrophage. IL-12-dependent macrophage infiltration enhances the CAR-T effect with synergistic effects *via* multiple immune response pathways. Moreover, given the pro-inflammatory subtype, IL-12-dependent macrophages can directly kill tumor cells *via* TNF-α secretion. Another study wherein IL-12 was directly injected into mice with lung cancer showed that the IL-12 injection reduced IL-10 and TGF-β levels at the tumor site, which are considered to be signals of the M2 TAM/M phenotype, and increased the expression of IL-6 and TNF-α (96). These studies suggest that TAM/M reprograming helps enhance the anti-tumor effect of CAR-T therapy by altering the immune inhibition environment created by TAM/Ms (**Figure 3B**).

In anti-PD-1 therapy, various studies have validated that the therapeutic effect of anti-PD-1 blockade in GBM is mediated *via* the innate immune system, rather than by CD8+ T cells. Anti-PD-1 immunologically modulates innate immunity in the glioma microenvironment, which is likely a key mode of activity (97, 98). In a multicohort, phase 1b study, an anti-PD-1 immunotherapy using pembrolizumab demonstrated durable antitumor activity among a subset of PD-L1-positive, recurrent glioblastoma patients (99). Furthermore, previous studies have demonstrated that combination therapy with anti-PD-1 improves GBM outcomes. Saha et al. (100) demonstrated that the triple combination of anti-CTLA-4, anti-PD-1, and G47Δ-mIL12 healed most mice in the two glioma models. This treatment is related to macrophage influx and M1-like polarization, along with increased T effector to T regulatory cell ratios. In addition, CD4+ and CD8+ T cells, as well as macrophages, are required for synergistic curative activity. Wu et al. determined that combination immunotherapy comprising anti-CXCR4 and anti-PD-1 provides a survival benefit in GBM through immune cell modulation of the tumor microenvironment (101). These studies have shown that targeting differentiation signals of TAM/Ms can help reverse TAM/M functions from an immunosuppressive to an anti-tumor state, which disturbs the M2 TAM/M function and changes CD8+ T cell homeostasis. Overall, this enhances the cytotoxic effect of CD8+ T cells against GBM cells.

### TAM/M-Derived Factors

In general, interaction between TAM/Ms and CD8+ T cells are thought to be counteractive. To activate the suppressive anti-tumor ability of CD8+ T cells, there are other potential pathways that need to be explored. A recent study demonstrated that the Fcγ receptor (FcγR), an immunoglobin receptor, has potential as an intervening target to renew TAM/M and CD8+ T cell crosstalk. In a colon cancer model, injection of anti-PD-1 antibodies was hypothesized to target PD-1 on the surface of CD8+ T cells, which helps reverse immunosuppression. However, anti-PD-1 antibodies were found to be recaptured by TAM/Ms *via* FcγR, which indicates another mechanism by which TAM/Ms help maintain an immunosuppressive microenvironment in immune checkpoint therapy (102). Further research has shown that a combination of anti PD-1 antibody and anti-FcγR antibody results in a much higher tumor inhibition rate than monotherapy with anti PD-1 antibody.

Another promising target is arginase-1 (ARG-1), which is known to be overexpressed in the M2 phenotype of macrophages as a potential biomarker (103). ARG-1 is an L-arginine enzyme that has been proven to have the capability of suppressing CD8+ T cell function through L-arginine depletion (66). High ARG-1-expressing TAM/Ms that are separated from the orthotopic pulmonary fibrosarcoma can also inhibit the proliferation of T cells (104, 105). An *in vivo* study demonstrated that treatment with anti-CTL4 antibody, in combination with the ARG-1 inhibitor CB-1158, significantly inhibited tumor growth in a lung metastasis mouse model (106). Likewise, similar results were reported with co-treatment with anti-PD-1 antibody and CB-1158 in a mouse model of colon cancer (92).

**Figure 4:**
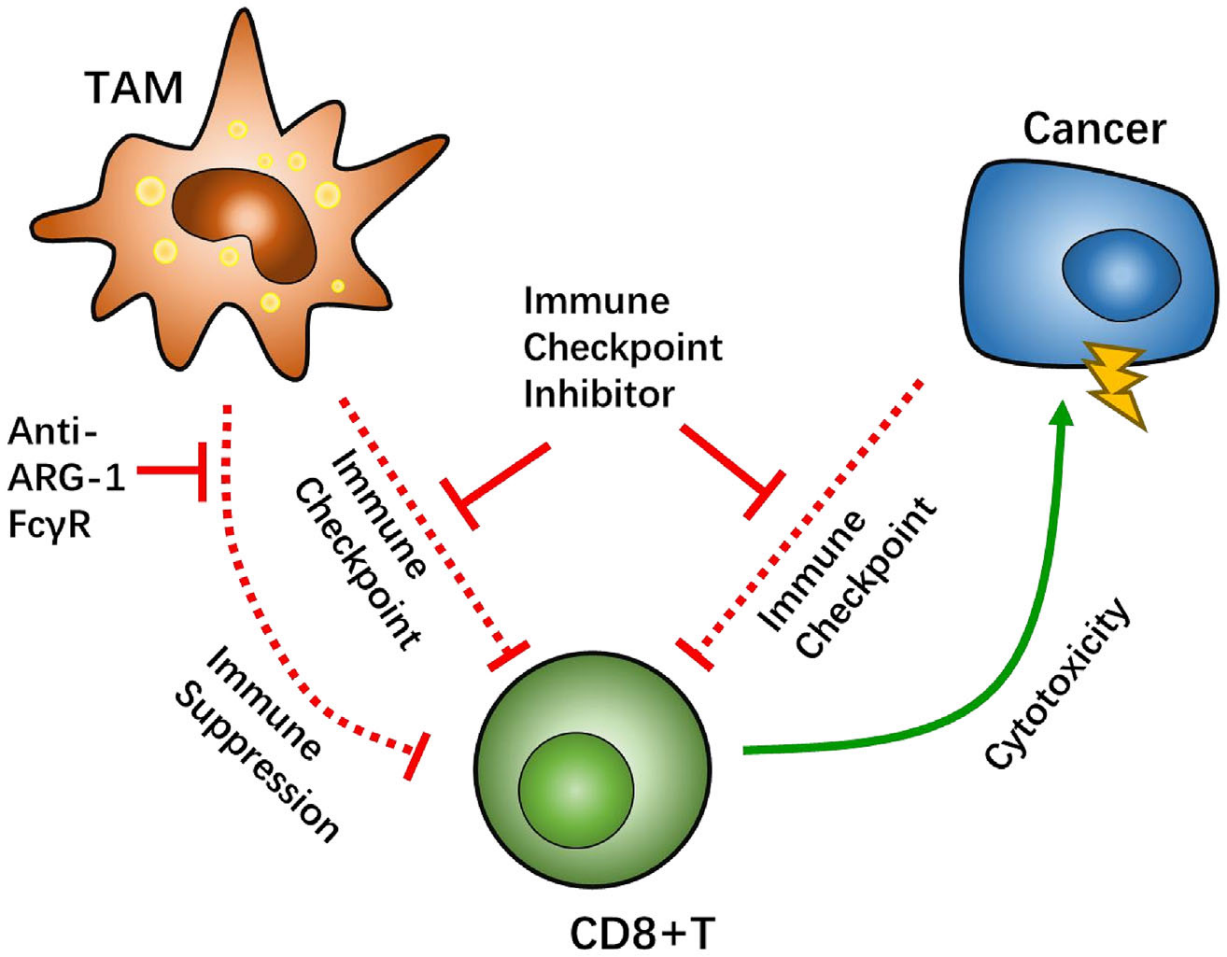
Tumor-associated macrophages (TAMs) factor as potential targets to overcome CD8-positive (CD8+) T cells suppression. The Fcγ receptor (FcγR) has been tested *in vivo* as a target for TAMs-CD8+ T cell interaction. Arginase-1 (ARG-1) can regulate CD8+ T cell metabolism *via* L-arginine depletion.

Signal transducers and activators of transcription 3 (STAT3) pathway is not only a growth controlling factor of GBM cells (107) but also a signal that regulates transcription of CD86 and CD80, as well as several macrophage-related target genes (108). Injecting small interfering RNA (siRNA) targeting STAT3 in GBM-bearing mice demonstrated promising results with respect to TNF-α-related macrophages, thereby altering the immunosuppressive environment to an optimal survival benefit (109) (**Figure 4**).

Interestingly, GBM and M2-like immunosuppressive macrophages promote angiogenesis, whereas M1-like pro-inflammatory macrophages suppress angiogenesis, which is known as “inflammation-driven angiogenesis”. Furthermore, soluble immunosuppressive cytokines, including predominantly TGF-β1 (which inhibits CD8+T cell), and surface integrin (αvβ3) endothelial-macrophage interactions are required in inflammation-driven angiogenesis. Therefore, dual αvβ3 and cytokine receptor (TGFβ-R1) blockade suppresses GBM tumor neovascularization by simultaneously targeting macrophage-associated immunosuppression and endothelial-macrophage interactions (110). Additionally, targeting TGF-β1 and TGF-β2 may improve intratumoral T cell infiltration and thus, enhance the effectiveness of immunotherapeutic approaches in GBM (111).

## Conclusion and Perspectives

There has been significant discussion regarding the immune system in the CNS over the past decade. However, the real situation has not been clearly described. The crosstalk between TAM/Ms and CD8+ T cells is still very important in the GBM immune response. Many potential targets have been studied for improving the prognosis of GBM patients and for disease prevention. Several studies have been successfully translated into clinical practice with promising results. Nevertheless, GBM, a malignant disease of the CNS, is still hard to treat. More studies are required to uncover the mechanisms associated with the symphonic immune interactions in GBM.

In this review, we discussed the role of TAM/Ms and CD8+ T cells in GBM, as well as the interactions between them. TAM/Ms in GBM are dominant in their anti-inflammatory phenotype, which is the main effector responsible for creating an immunosuppressive GBM microenvironment. TAM/Ms are able to inhibit the cytotoxic effect of CD8+ T cells through a multitude of biological pathways and cytokines. This suppressive process not only appears amidst development of GBM but also appears during the application of GBM treatment.

There are potential therapeutic targets that underlie the crosstalk between TAM/Ms and CD8+ T cells. Sabotaging TAM/M recruitment and migration can cause the depletion of TAM/Ms, which helps revive the cytotoxic effect of CD8+ T cells that can help lead the fight against GBM cells. CCL2 and CSF-1 inhibitor have shown significant potential for further clinical evaluation to improve CTL-related immune therapies, such as immune checkpoint inhibitor therapy, and CAR-T therapy. To reverse the immunosuppressive situation in the GBM tissue, reprograming TAM/Ms can help shift TAM/Ms from an anti-inflammatory phenotype, toward a pro-inflammatory phenotype, enhancing the CD8+ function of CTLs. IL-10, IL-12, and PI3Kγ have a significant function in this process. Furthermore, biological agents can be used to boost suitable therapies. Moreover, novel TAM factors have been tested with optimal results, as anti-tumor targets. Interactions between TAM/Ms and CD8+ T cells were considered to have a decisive power in GBM physiology, as well as immune therapy for GBM. Finally, the underlying targets for GBM therapy need to be further studied to clinically improve the outcomes of GBM patients.

## Data Availability Statement

The original contributions presented in the study are included in the article/supplementary material, further inquiries can be directed to the corresponding authors.

## Author Contributions

YZ and AS conceptualized the research project. ST, XL, AS, and JZ wrote the paper and made the original figures. ST, HW, YY, JQ, SH, and YW critically revised the texts and figures. AS, YD, and YZ supervised the research and led the discussion. All authors contributed to the article and approved the submitted version.

## Funding

This work was funded by National Natural Science Foundation of China (81701144) and Zhejiang Provincial Natural Science Foundation of China (LQ19H160045).

## Conflict of Interest

The authors declare that the research was conducted in the absence of any commercial or financial relationships that could be construed as a potential conflict of interest.

## Publisher’s Note

All claims expressed in this article are solely those of the authors and do not necessarily represent those of their affiliated organizations, or those of the publisher, the editors and the reviewers. Any product that may be evaluated in this article, or claim that may be made by its manufacturer, is not guaranteed or endorsed by the publisher.

## Abbreviations

BBB, blood–brain barrier; CAR-T, chimeric antigen receptor T cell; CCL2, chemotactic protein-1; CD68, clusters of differentiation 68; CD8+, CD8 positive; CNS, central nervous system; CSF1R, colony-stimulating factor 1 receptor; CTL, cytotoxic lymphocytes; DAMPs, damage-associated molecular patterns; DC, dendritic cells; FAS, first apoptosis signal; FASL, first apoptosis signal ligand; FcγR, Fcγ receptor; FRβ,folate receptor β; GBM, glioblastoma multiforme; ICI, immune checkpoint inhibitor; IDO, indoleamine 2,3-dioxygenase; IFN-γ, interferon-gamma; iNOs, inducible NO synthase; MARCO, macrophage receptor with collagenous structure; MCH-1, histocompatibility complex class I; PAMPs, pathogen-associated molecular patterns; TAMs, tumor associated macrophages; TAM/Ms, tumor-associated microglia/macrophages; TGF-β, tumor growth factor β; Treg, regulation T cells; VISTA, V-domain Ig suppressor of T cell activation.
